# Towards elimination of mother-to-child transmission of HIV in Ghana: an analysis of national programme data

**DOI:** 10.1186/s12939-016-0300-5

**Published:** 2016-01-13

**Authors:** P. Dako-Gyeke, B. Dornoo, S. Ayisi Addo, M. Atuahene, N. A. Addo, A. E. Yawson

**Affiliations:** Department of Social and Behavioral Sciences, College of Health Sciences, University of Ghana School of Public Health, Legon, Accra Ghana; National AIDS/STI Control Programme, Ghana Health Service, Korle- Bu, Accra Ghana; Department of Population Family and Reproductive Health, University of Ghana School of Public Health, Legon, Accra Ghana; Departmentof Community Health, University of Ghana School of Public Health, College of Health Sciences, Korle-Bu, Accra Ghana

**Keywords:** HIV prevalence, mother-to-child HIV transmission, HIV testing, Antiretroviral therapy, Ghana

## Abstract

**Background:**

Despite global scale up of interventions for Preventing Mother to child HIV Transmissions (PMTCT), there still remain high pediatric HIV infections, which result from unequal access in resource-constrained settings. Sub-Saharan Africa alone contributes more than 90 % of global Mother-to-Child Transmission (MTCT) burden. As part of efforts to address this, African countries (including Ghana) disproportionately contributing to MTCT burden were earmarked in 2009 for rapid PMTCT interventions scale-up within their primary care system for maternal and child health. In this study, we reviewed records in Ghana, on ANC registrants eligible for PMTCT services to describe regional disparities and national trends in key PMTCT indicators. We also assessed distribution of missed opportunities for testing pregnant women and treating those who are HIV positive across the country. Implications for scaling up HIV-related maternal and child health services to ensure equitable access and eliminate mother-to-child transmissions by 2015 are also discussed.

**Methods:**

Data for this review is National AIDS/STI Control Programme (NACP) regional disaggregated records on registered antenatal clinic (ANC) attendees across the country, who are also eligible to receive PMTCT services. These records cover a period of 3 years (2011–2013). Number of ANC registrants, utilization of HIV Testing and Counseling among ANC registrants, number of HIV positive pregnant women, and number of HIV positive pregnant women initiated on ARVs were extracted. Trends were examined by comparing these indicators over time (2011–2013) and across the ten administrative regions. Descriptive statistics were conducted on the dataset and presented in simple frequencies, proportions and percentages. These are used to determine gaps in utilization of PMTCT services. All analyses were conducted using Microsoft Excel 2010 version.

**Results:**

Although there was a decline in HIV prevalence among pregnant women, untested ANC registrants increased from 17 % in 2011 to 25 % in 2013. There were varying levels of missed opportunities for testing across the ten regions, which led to a total of 487,725 untested ANC clients during the period under review. In 2013, Greater Accra (31 %), Northern (27 %) and Volta (48 %) regions recorded high percentages of untested ANC clients. Overall, HIV positive pregnant women initiated onto ARVs remarkably increased from 57% (2011) to 82 % (2013), yet about a third (33 %) of them in the Volta and Northern regions did not receive ARVs in 2013.

**Conclusions:**

Missed opportunities to test pregnant women for HIV and also initiate those who are positive on ARVs across all the regions pose challenges to the quest to eliminate mother-to-child transmission of HIV in Ghana. For some regions these missed opportunities mimic previously observed gaps in continuous use of primary care for maternal and child health in those areas. Increased national and regional efforts aimed at improving maternal and child healthcare delivery, as well as HIV-related care, is paramount for ensuring equitable access across the country.

## Background

Despite global scale up of interventions for Preventing Mother to Child HIV Transmissions (PMTCT), there still remain high pediatric HIV infections, which result from unequal access in resource-constrained settings [1–4]. In 2008, approximately 350,000 new mother-to-child HIV transmissions (MTCT) occurred in low- and middle-income countries [1], with Sub-Saharan Africa contributing more than 90 % [5]. To help reduce MTCT by 50 %, African countries (including Ghana) disproportionately contributing to the burden were earmarked for rapid PMTCT intervention scale-up within their primary care system for maternal and child health [1]. Ghana in 2009 recorded high HIV prevalence (2.9 %) among antenatal clinic attendees and about 3700 new infant HIV infections [6] which accounted for approximately 15 % of all HIV transmissions in the country [7].

Mother to child HIV transmissions can be prevented through effective implementation of multiple interventions within the primary care system for maternal and child health; these are categorized into four prongs by the United Nations - (1) primary prevention of HIV infection among women of childbearing age; (2) preventing unintended pregnancies among women living with HIV; (3) preventing HIV transmission from women living with HIV to their infants and (4) providing appropriate treatment, care and support to mothers living with HIV, their children and families [1, 8]. Through its rapid advice guidelines, WHO puts particular emphasis on the third prong, recommending treatment of eligible HIV-positive pregnant women with lifelong antiretroviral therapy (ART), by proposing two equivalent options of highly effective prophylaxis to HIV positive pregnant women who do not need ART for their own health [9]. These preventive interventions have been proven to be effective in reducing MTCT rate to about 2 % in developed countries [10, 11].

Rather than a standalone programme, WHO recommends integration of PMTCT interventions with standard primary care for maternal, neonatal and child health (MNCH) programmes [1]. In line with this, Ghana promotes MNCH-PMTCT integration either at a single point of access (unified) or through use of referrals [4, 12]. This integration is executed at national (tertiary), regional, district health centre level facilities, in both public and private health facilities, across the 10 administrative regions in the country. With further decentralization to include Community Based Health Planning Services (CHPS) sites, the number of PMTCT centres is known to have increased from 135 in 2005 to 1174 in 2011 [12]. Preventive interventions such as HIV testing and counseling, treatment, especially provision of antiretroviral therapy, and continuum of care are all integral parts of regular antenatal, labor and delivery, as well as postpartum services [3, 13].

Within high MTCT burden contexts, PMTCT-MNCH integration is used as a vehicle for ensuring equitable and universal access for women, since majority (79 %) of pregnant women are known to attend antenatal clinics [1]. Although, equitable access has been a key WHO PMTCT strategic vision (2010–2015) distribution of PMTCT interventions has been quite uneven in resource-limited settings [1, 4, 10]. For instance, in 2009 PMTCT coverage was below 50 % in approximately half of the high MTCT burden countries [5] and only a quarter (26 %) of pregnant women in low- and middle-income countries were estimated to have tested for HIV [5]. Moreover, estimates indicate that in 2012,only 58 % of pregnant women who needed antiretroviral therapy for their own health received HIV treatment, which is below the 64 % treatment coverage for other eligible adults [2]. Furthermore, in a very recent gap analysis report, WHO estimates that approximately 1.3million women still do not have access to PMTCT interventions within its 21 priority countries [14]. Gaps in access must be addressed considering the estimated high risk (25–40 %) of MTCT if an HIV positive pregnant woman does not receive any antiretroviral medicines [15]. Greater efforts are required to close gaps in access as new analysis suggests that a 10 % increase in treatment coverage is likely to reduce new infections by 1 % [14].

Previous studies have identified structural, socioeconomic, and cultural factors as key limitations to PMTCT access in resource limited settings [1, 10, 16]. While primary health care system deficiencies lead to missed opportunities and low HIV testing among pregnant women [16–18], other larger socio-cultural factors including low PMTCT knowledge, negative perceptions, stigma, fear and lack of support also inhibit use of PMTCT services in resource limited-countries [17–20]. Moreover, in the event of PMTCT-MNCH integration, already existing maternal healthcare delivery challenges in low-income countries may be adversely facilitating unequal PMTCT access in some contexts. Similar to maternal and child health service packages, PMTCT interventions are supposed to be implemented in a comprehensive and continuous manner. Unfortunately, observed gaps in the continuous use of recommended facility-based maternal health care services, such as low four-time antenatal visits (55 %) and low skilled birth attendance (50 %) in WHO African Region may limit equitable reach of PMTCT interventions among the target population [21].

In assessing extent to which WHOs strategic vision of equitable access to PMTCT interventions (2010–2015) has been achieved, there is need to examine distribution of use, especially in countries belonging to the 21 Global Plan [14]. Countries are encouraged to use local epidemiological data to show gaps in regional and district level coverage and in the process identify areas that are being left behind. In this regard, we reviewed records in Ghana, on ANC registrants eligible for PMTCT services to describe regional disparities and national trends in key PMTCT indicators. We also use this review to assess distribution of missed opportunities for testing pregnant women and treating those who are HIV positive across the country. Implications for achieving the target of 90 % reduction in mother-to-child transmissions by 2015 are also discussed. Extensive examination of current PMTCT programmes is critical to identifying implementation gaps and to suggest context-specific interventions that can improve access among eligible women of reproductive age.

## Methods

To describe performance on key PMTCT indicators, this study reviewed National AIDS/STI Control Programme (NACP) dataset. These were region-disaggregated records on pregnant women registered at the various antenatal clinics across the country, who are eligible to receive PMTCT services (i.e., all pregnant women who register for antenatal services are provided HIV testing with the option to ‘opt out’. Those who test positive for HIV are enrolled into care). These records cover 2011–2013. The National AIDS/STI Control Programme is responsible for coordination and implementation of HIV and AIDS- related aspects of the Ghana Health Strategic Framework. Implementation is managed by the Disease Control and Prevention Department of the Public Health Directorate of the Ghana Health Service. NACP sources and collates computerized HIV and AIDS- related service provision data from community health centers, district hospitals, regional hospitals and teaching hospitals throughout the country, every quarter. From these records, NACP generates a comprehensive national dataset that covers PMTCT service provision across all ten administrative regions in Ghana.

### Data handling

National level Programme Data generated by the NACP are treated with a high level of confidentiality. Unique identifiers and codes are employed to de-personify the records of clients and are used for computer-based data entry. Computerized records of national programme data are kept in password-protected files accessible to designated national programme officers only.

### Outcome measures and data analysis

In order to describe regional disparities and national trends key PMTCT indicators including number of ANC registrants, number tested for HIV (HTC) among ANC registrants, number of ANC registrants who tested HIV positive and number of HIV+ pregnant women who were initiated on to ARVs were extracted. Trends were examined by comparing these indicators (number registered at ANC, number receiving HTC, number who tested positive, number initiated on ART) over time (2011–2013). In addition, a detailed analysis of geographic (regional) location of pregnant women receiving PMTCT services from 2011 to 2013 was conducted. Furthermore, missed opportunities for testing ANC registrants for HIV was calculated by determining percentage of ANC registrants who were not tested for HIV each year. Also percent change between 2011 and 2013 was estimated. Additionally, missed opportunities to treat HIV positive pregnant women was calculated by determining percentage of pregnant HIV positive women who were not initiated on to ARVS.

Also percent change between 2011 and 2013 was obtained. Percent (%) change in the number of PMTCT cases untested or not initiated between 2011 and 2013 was estimated as follows:

$$ \%\  Change=\left(\frac{\left[\left( PMTC{T}_{present}\right)\times p\right]-\left[\left( PMTC{T}_{past}\right)\times p\right]}{\left[\left( PMTC{T}_{present}\right)\times p\right]}\right)\times 100 $$

Where, *PMTCT*_*present*_ is the present number of cases (in 2013), *PMTCT*_*past*_ is the past number of cases (in 2011) and *p* is the proportion of cases untested for HIV or not initiated on ARVs in the corresponding year.

Data analysis of outcome measures was by descriptive statistics (simple frequencies, proportions and percentages) to determine gaps in utilization of PMTCT services. Also, 95 % Confidence Interval have been calculated for all proportions indicated in Tables 1 and 2. All analyses were conducted using Microsoft Excel 2010 version.

**Table 1:** Antenatal clinic (ANC) registrants not tested across the country (2011–2013)

Number of ANC Registrants and Percent Untested				
		Year		
	2011	2012	2013	2011–2013
Region	ANC Registrants (% Untested) (95 % CI)	ANC Registrants (% Untested) (95 % CI)	ANC Registrants (% Untested) (95 % CI)	Percent Change (Untested)^a^
Ashanti	122,708 (12 %) (11.8, 12.2)	110,438 (22 %) (21.8,22.4)	106,189 (19 %) (18.8,19.2)	27 %
Brong Ahafo	66,842 (15 %) (14.7, 15.3)	64,515 (15 %) (14.7,15.3)	66,965 (16 %) (15.7,16.3)	6.4 %
Central	58,459 (4 %) (3.8, 4.2)	64,429 (14 %) (13.7,14.3)	55,763 (27 %) (26.6,27.4)	84.4 %
Eastern	89,341 (11 %) (3.8, 4.2)	90,648 (30 %) (29.7,30.3)	86,018 (23 %) (22.7,23.2)	50.3 %
Greater Accra	145,235 (30 %) (29.8,30.2)	150,727 (36 %) (35.8,36.4)	152,730 (31 %) (30.8,31.2)	7.9 %
Northern	99,195 (13 %) (12.8,13.2)	86,436 (15 %) (14.8,15.2)	47,730 (27 %) (26.6,27.4)	−.06 %
Upper East	36,188 (7 %) (06.7,07.3)	34,172 (4 %) (03.8,04.2)	32,140 (3 %) (02.8,03.2)	−162 %
Upper West	24,407 (16 %) (15.1,16.0)	21,940 (10 %) (09.6,10.4)	21,941 (10 %) (09.6,10.4)	−77.9 %
Volta	73,038 (43 %) (42.6,43.4)	72,410 (42 %) (41.6,42.4)	70,775 (48 %) (47.6,48.4)	7.5 %
Western	57,542 (6 %) (05.8,06.2)	40,803 (18 %) (17.6,18.4)	65,366 (17 %) (16.7,17.3)	68 %
TOTAL	772,955 (17 %) (16.9,17.1)	736,518 (24 %) (23.9,24.1)	705,617 (25 %) (24.9,25.1)	25 %

^a^Negative sign for the percent change indicates reduction in the number of HIV Positive Pregnant Women not Initiated on ARVs from 2011 to 2013

**Table 2:** HIV positive pregnant women not initiated on ARVs in the regions (2011–2013)

HIV Positive Pregnant Women Not Initiated on ARVs in All Regions				
	Year			
	2011	2012	2013	2011–2013
Region	HIV+ ANC Clients (% Not Initiated on ARVs) (95 % CI)	HIV+ ANC Clients (% Not Initiated on ARVs) (95 % CI)	HIV+ ANC Clients (% Not Initiated on ARVs) (95 % CI)	Percent Change (Not initiated on ARVs)^a^
Ashanti	3391 (68 %) (66.4,69.6)	2215 (31 %) (29.1,32.9)	1508 (19 %) (17.0,21.0)	−704 %
Brong Ahafo	1414 (22 %) (19.9,24.2)	1348 (13 %) (11.2,14.9)	1101 (4 %) (37.1,42.9)	−606 %
Central	619 (31 %) (27.2,34.7)	725 (32 %) (28.6,35.5)	387 (28 %) (23.5,32.7)	−77 %
Eastern	2052 (16 %) (14.4,17.6)	1967 (17 %) (15.3,18.7)	1633 (14 %) (12.3,15.7)	−43 %
Greater Accra	2268 (35 %) (33.0,37.0)	1971 (4 %) (37.8,42.2)	1789 (29 %) (26.9,31.1)	−53 %
Northern	417 (73 %) (68.4,77.1)	285 (2 %) (15.5,25.1)	231 (33 %) (26.9,39.4)	−299 %
Upper East	240 (45 %) (38.6,51.5)	253 (27 %) (21.5,32.8)	242 (−22 %) (16.9,27.6)	−102 %
Upper West	183 (19 %) (13.2,25.0)	171 (52 %) (44.2, 59.7)	167 (−31 %) (23.7,38.1)	32.8 %
Volta	1555 (58 %) (55.4,60.4)	684 (14 %) (43.7,59.2)	758 (33 %) (29.6,36.5)	−260 %
Western	1492 (42 %) (39.4,44.5)	770 (15 %) (12.5,17.7)	1093 (17 %) (14.7,19.3)	−237 %
TOTAL	13,631 (43 %) (42.2,43.8)	10,389 (17 %) (16.3,17.7)	8909 (18 %) (17.2,18.8)	−265 %

^a^Negative sign for the percent change indicates reduction in the number of HIV Positive Pregnant Women not Initiated on ARVs from 2011 to 2013

### Ethical issues

Clearance for this analysis was provided by the National Programme Manager and officers of the NACP, with consent from the Ghana Health Service.

## Results

### ANC registrants tested for HIV (2011–2013)

During the period under review (2011–2013) a total of 2,215,090 pregnant women registered at various antenatal clinics, and were eligible for PMTCT services (i.e., all pregnant women who register for antenatal services are provided HIV testing with the option to ‘opt out’. Those who test positive for HIV are enrolled into care). Generally, there was a downward trend in the number of pregnant women that registered for ANC services from 772,995 in 2011 to 705,617 in 2013 (Table 1). Overall, 78 % of ANC clients were tested for HIV across the country (2011–2013). There was an increase in untested ANC clients from 17 % (2011) to 25 % (2013) with a 25 % change (Table 1). The data showed that the percentage of untested ANC clients for each year under review was consistently high in the Volta region (2011, 31,406; 2013, 33,972), with an average of 44 % missed opportunities to test ANC registrants (Table 1). Similarly, Greater Accra region recorded an average of 32 % ANC untested clients. Although, Western region recorded a low percentage (6 %, 3452) of untested ANC clients in 2011, there was a three-fold increase (18 %) in 2012 and a 68 % change by 2013. Also, Upper East region consistently recorded an average of 4 % untested ANC clients. Northern and Central regions, each had 27 % untested ANC clients in 2013, which was relatively higher than what was recorded in previous years (Table 1). The lowest percentage of untested ANC clients in 2011 was in the Central region (4 %), with 84 % change between 2011 and 2013 (Table 1). Also, Upper East region recorded the lowest percentage of untested clients in both 2012 (4 %) and 2013 (3 %).

### HIV positive ANC clients (2011–2013)

**Fig. 1:**
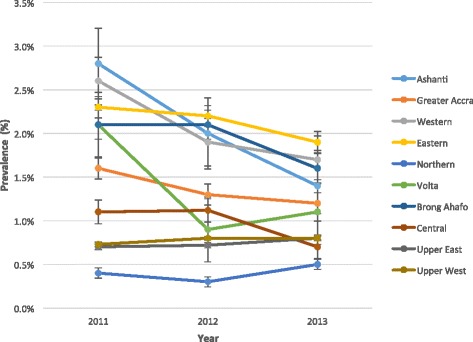
HIV positive ANC clients (2011–2013)

Generally, there was a downward trend in HIV prevalence among ANC Clients in all ten regions between 2011 and 2013 (Fig. 1). In 2011, five regions (Ashanti, Eastern, Western, Volta and Brong Ahafo) recorded about 2 % HIV prevalence among its ANC clients. However, in the year 2012 only three regions (Ashanti, Eastern and Brong Ahafo) recorded about 2 % HIV prevalence among ANC clients Fig. 1). In contrast, the Northern, Upper East and Upper West Regions consistently recorded HIV prevalence, which was below 1 % in each of the years under review.

### HIV Positive pregnant women not initiated on ARVs (2011–2013)

There was a decline in HIV positive ANC clients who were not initiated on ARVs from 43 % in 2011 to 18 % in 2013. This reduction is also indicated by the negative percent change recorded for all 10 regions (Table 2). In 2011, more than half of pregnant women who tested positive for HIV in three regions (Ashanti, 68; Northern, 73; and Volta, 58 %) were not initiated on ARVs. Two regions consistently recorded substantial declines in HIV positive clients not initiated on ARVs between 2011 and 2013; Ashanti (68 %; 19 %) and Brong Ahafo (22 %; 4 %). Also, two regions (Northern and Volta regions) each recorded the highest of 33 % ANC HIV positive clients not put on ARVs in 2013. These were followed by, Greater Accra (29 %) and Central (28 %) regions. Also the two Upper regions also recorded negative percentages for HIV positive clients not put on ARVs.

## Discussion

Globally, there is a comparatively low anti-retroviral therapy use among pregnant women living with HIV than what pertains among other eligible adults [2]. This undermines the global target of eliminating mother-to-child HIV transmissions by 2015 [1, 2]. Although findings from this review demonstrate remarkable improvement in the percentage of HIV positive pregnant women who received ARVs there remains a disproportionate use of PMTCT interventions across the ten administrative regions in the country. Our review shows a quarter of pregnant women did not receive HIV testing in 2013. In addition, three regions (Volta, Greater Accra, and Central) recorded high un-tested rates among pregnant women, as well as low initiation of HIV positive pregnant women unto ARVs in 2013. This is unfortunate because research shows that women refusing HIV testing are likely to be HIV infected than those accepting it [22]. Also, there are concerns about effectiveness of the PMTCT programme in ensuring universal access through routine HTC at the antenatal clinics [23]. Wettstein and colleagues demonstrate through a systematic review that use of the opt-out testing approach leads to high HTC (94 %) among pregnant women than the use of the opt-in testing (58 %) [8]. Although, Ghana is currently practicing the opt-out approach, we are yet to capture a high percentage of pregnant women for HTC.

Pragmatically, we believe that differentials in use of PMTCT interventions, especially in some regions, may only be a re-enactment of observed gaps in the continuous use of facility-based maternity care, especially skilled assistance during delivery in the country [24, 25]. Ghana promotes PMTCT-MNCH integration, which calls for effective management, resource mobilization and coordination of all PMTCT interventions with maternal and child healthcare delivery packages. Unfortunately, such efforts are limited by deficiencies characterizing maternal healthcare delivery and utilization across the country. Whereas 95 % of pregnant Ghanaian women receive some form of antenatal care, only about three-quarters pay the WHO recommended four-time ANC visits, with only 57 % births occurring in health facilities [25]. Also, statistics show that less than half (48 %) of Ghanaian women utilize the entire maternal health care package starting from antenatal, through labor and delivery to postnatal services [25]. Since PMTCT interventions are multiple and must be offered in a continuous manner within the facility-based healthcare system, limitations in use of maternity care will negatively impact implementation of PMTCT interventions, as well.

Furthermore, the possible link between the extent of missing opportunities for offering PMTCT interventions and gaps in continued use of facility-based maternity care is particularly evident in two regions in Ghana. Low HIV testing and low initiation of HIV positive women unto ARVs in the Volta and Central regions were similar to observed trends in use of antenatal care and facility-based deliveries in these areas, as reported by the Ghana Demographic Health Survey [25]. For instance, the Volta region recorded 91 and 54 % antenatal visits and facility-based deliveries, respectively; whilst Central region recorded 92 and 52 %, antenatal visits and facility-based deliveries, respectively [25]. It is worthy to note that in all instances, records for use of HIV testing, initiation of HIV positive women unto ARVs, antenatal visits, as well as facility-based deliveries in these two regions were below the national average. Interestingly, the Northern region, recording the lowest facility-based deliveries [25] also had a high percentage of HIV positive women not put on ARVs in 2013. Although, Greater Accra region also recorded disproportionate use of some PMTCT interventions antenatal visits and hospital deliveries are high. This may be explained by the fact that the region hosts the capital city (Accra) and is much more cosmopolitan with several health facilities available. There by provision of maternity care may be of a wider reach within the region.

In this situation PMTCT-MNCH linkage may serve as double indicator for measuring extent to which equitable access can be achieved using already existing maternal healthcare delivery structures. On one hand, it highlights potentials for effectively utilizing already established maternal health delivery structures, which are useful for implementing PMTCT interventions [4]. However, it can also expose potential weaknesses in the maternal health services delivery system and how they may be overburdened; such deficiencies are often illustrated by high maternal deaths. In each scenario, countries should endeavor to strengthen health structures for delivering maternity care and ultimately ensure equitable access among pregnant women. Also, it is key to understand that use of interventions for preventing mother to child HIV transmissions is largely dependent not only on access to facility-based prenatal, obstetric, and postnatal care, but also the reliability of services delivered [26]. Furthermore, HIV-related stigmatization is known to undermine PMTCT efforts within several sub-Saharan African contexts [27, 28]. For fear of disclosure some women tend to opt out of PMTCT programmes, which results in missed opportunities for being tested and receiving treatment and care [28, 29] In Cote d’Ivoire a study found that unpleasant healthcare provider attitudes as well as denial of test results were factors that could inhibit some HIV+ pregnant women from being initiated on to ARVs [30].

Regardless of regional variations, there was a remarkable increase in number of HIV positive pregnant women initiated onto ARVs annually across the country. This finding is similar to WHO’s 2012 estimate of 90 % coverage in some high priority countries (Ghana, Botswana, Namibia and Zambia) known to have already achieved the global goal [2]. Also, some regions (Upper East and Upper West) recorded excesses, which probably imply that services are being extended to clients from the other regions. All these are indicative of numerous scale-up attempts across the country which are in accordance with global efforts for addressing the needs of countries with high HIV prevalence among pregnant women [1].

Although this review gives detailed analysis on the country’s performance on key PMTCT indicators it has some limitations. This dataset is based on routine service provision records that may sometimes be incomplete. Also, since this data does not include demographic information on clients it is difficult to determine double counting. We also acknowledge data on women accessing PMTCT through private sector and delivering at home without skilled birth attendant might not be captured in national programme data. However, considering NACP’s rigorous data collection processes these limitations might not necessarily alter the key findings of this study.

## Conclusion

Despite notable progress in the provision of PMTCT services across the country, major challenges to both scaling up services and increasing coverage of PMTCT services remain. Missed opportunities to test pregnant women and initiate those who are HIV+ onto ARVs across all regions pose challenges to the quest to eliminate MTCT in Ghana. Additionally, this study highlights critical gaps that remain for care provided during pregnancy and time of birth when the risk of mortality is highest for mother and child. Regional differences also draw attention to need to specifically focus on some key regions to help address the unequal gaps in performance on selected indicators. In this regard, we recommend that regions that continue to miss opportunities to provide HIV testing, treatment and care to pregnant women should be assessed on the feasibility of integrating the PMTCT programme with MNCH services in their contexts. Also, there should be a follow-up mechanism that allows facilities to identify, contact and track pregnant women who are not tested, as well as HIV+ clients who do not receive treatment. Increased national and regional efforts in maternal and child HIV-related care are paramount and should be pursued by the Ghana Health Service through the National AIDS/STI Control Programme and all stakeholders in the HIV arena in Ghana.

## Abbreviations

ANC: antenatal clinic
ARV/ART: anti-retroviral therapy
CHPS: community based health planning services
GAC: Ghana AIDS Commission
HTC: HIV testing and counseling
MNCH: maternal, neonatal and child health
MTCT: mother to child transmission
NACP: National AIDS/STI Control Programme
PMTCT: preventing mother to child hiv transmission
WHO: World Health Organization
